# The Relationship between RTS,S Vaccine-Induced Antibodies, CD4^+^ T Cell Responses and Protection against *Plasmodium falciparum* Infection

**DOI:** 10.1371/journal.pone.0061395

**Published:** 2013-04-16

**Authors:** Michael T. White, Philip Bejon, Ally Olotu, Jamie T. Griffin, Eleanor M. Riley, Kent E. Kester, Christian F. Ockenhouse, Azra C. Ghani

**Affiliations:** 1 MRC Centre for Outbreak Analysis and Modelling, Department of Infectious Disease Epidemiology, Imperial College London, London, United Kingdom; 2 Kenya Medical Research Institute, Kilifi, Kenya; 3 Centre for Clinical Vaccinology and Tropical Medicine, University of Oxford, Oxford, United Kingdom; 4 Department of Immunology and Infection, Faculty of Infectious and Tropical Diseases, London School of Hygiene and Tropical Medicine, London, United Kingdom; 5 Walter Reed Army Institute of Research, Silver Spring, Maryland, United States of America; World Health Organization, Switzerland

## Abstract

Vaccination with the pre-erythrocytic malaria vaccine RTS,S induces high levels of antibodies and CD4^+^ T cells specific for the circumsporozoite protein (CSP). Using a biologically-motivated mathematical model of sporozoite infection fitted to data from malaria-naive adults vaccinated with RTS,S and subjected to experimental *P. falciparum* challenge, we characterised the relationship between antibodies, CD4^+^ T cell responses and protection from infection. Both anti-CSP antibody titres and CSP-specific CD4^+^ T cells were identified as immunological surrogates of protection, with RTS,S induced anti-CSP antibodies estimated to prevent 32% (95% confidence interval (CI) 24%–41%) of infections. The addition of RTS,S-induced CSP-specific CD4^+^ T cells was estimated to increase vaccine efficacy against infection to 40% (95% CI, 34%–48%). This protective efficacy is estimated to result from a 96.1% (95% CI, 93.4%–97.8%) reduction in the liver-to-blood parasite inoculum, indicating that in volunteers who developed *P. falciparum* infection, a small number of parasites (often the progeny of a single surviving sporozoite) are responsible for breakthrough blood-stage infections.

## Introduction

Malaria continues to pose a serious public health challenge, with an estimated 655,000 malaria associated deaths every year [1], despite the large scale roll out of insecticide treated nets across the globe [2] and the switch to treatment with highly efficacious artemisinin combination therapies [3]. An efficacious malaria vaccine would be an invaluable addition to the range of currently available malaria control interventions. The malaria vaccine candidate RTS,S, targeting the pre-erythrocytic stages of *Plasmodium falciparum,* has been shown to prevent malaria infection and clinical disease in Phase 2b field trials in infants [4]–[6], children [7], [8] and adults [9], [10] as well as more recently in a large Phase 3 trial underway in Africa [11]. RTS,S targets the circumsporozoite protein (CSP) and has been formulated with either of two different adjuvant systems; AS02 or AS01. In field trials where RTS,S/AS01 and RTS,S/AS02 have been directly compared, RTS,S/AS01 has been found to be more immunogenic [9], [12], [13].

Sporozoites inoculated into the skin via mosquito bite can be opsonised and immobilised by vaccine-induced anti-CSP antibodies as they migrate through tissue [14]. Sporozoites that reach the liver will invade hepatocytes where they undergo hepatic development. Hepatocyte invasion could potentially be prevented by anti-CSP antibodies [15]. Intracellular *Plasmodium* parasites can be targeted by vaccine-induced CSP-specific CD4^+^ T cells leading to killing of the infected hepatocyte [16], [17]. After approximately 6.5 days of hepatic development [18], [19], merozoites will be released into the blood circulation to begin the erythrocytic stage of infection. When released from the liver, merozoites undergo blood-stage replication causing an exponential increase in parasite numbers. Studies of early blood-stage *P. falciparum* infection in human volunteers have demonstrated that the smaller the liver-to blood inoculum, the longer the time taken for parasite density to reach a given threshold [20], [21].

Vaccination with RTS,S induces anti-CSP antibodies and CSP-specific CD4^+^ T cells that produce a mixture of cytokines (such as IL-2, TNF-α, IFN-γ) and may also express the co-stimulatory molecule CD40L [17], [22]. Protection from infection and clinical disease has been shown to be associated with both naturally-acquired and RTS,S induced anti-CSP antibodies [23], [24]. CSP-specific CD4^+^ T cells have been associated with protection from infection in RTS,S vaccinated children [25] and in children with naturally-acquired immunity [26]. Characterising precise immunological surrogates of protection in field trials is, however, complicated by heterogeneous exposure to malaria, temporal changes in immune markers, and interactions with naturally-acquired immunity [27], [28]. In contrast, challenge trials in malaria-naïve adults provide an ideal opportunity to investigate the dose-response relationship between immune markers and protection from infection as the infectious dose can be controlled and the timing known, there is no naturally-acquired immunity, and immune markers can be measured on the day of challenge.

Kester *et al* [29] undertook such a challenge study for RTS,S/AS01 and RTS,S/AS02 in malaria naïve adults. 52 volunteers were vaccinated with RTS,S/AS01 and 50 volunteers with RTS,S/AS02. 36 volunteers were recruited as controls and hence remained unvaccinated. 104 volunteers were challenged with the bites of five *P. falciparum* infectious *Anopheles stephensi* mosquitoes [30]. The efficacy of RTS,S/AS01 and RTS,S/AS02 against infection was estimated to be 50% (95% CI, 32.9%–67.1%) and 32% (95% CI, 17.6%–47.6%), respectively. Protected vaccine recipients had higher anti-CSP antibody titres (mean, 188 vs. 73 µg/mL; P<0.001), and higher numbers of CSP-specific CD4^+^ T cells per million CD4^+^ T cells (median, 963 vs. 308 CSP-specific CD4^+^ T cells; P<0.001) than unprotected vaccine recipients. The study also demonstrated significantly higher levels of anti-CSP antibody titres and numbers of CSP-specific CD4^+^ T cells in those vaccinated with RTS,S/AS01 compared to RTS,S/AS02. Here we re-analyze the data to investigate in detail the association between RTS,S-induced anti-CSP antibodies, CD4^+^ T cells and protection from infection using a biologically-motivated mathematical model of *P. falciparum* sprorozoite inoculation to estimate the probability of infection and the delay in onset of parasitemia due to vaccination. Our results provide insights into the likely mechanism of action of the RTS,S vaccine as well as providing a more generalised framework for assessing the efficacy of vaccines in early stage development.

## Methods

### Challenge Trial

Kester *et al* [29] evaluated the efficacy and safety of the RTS,S malaria vaccine when formulated with the AS01 and AS02 adjuvant systems in 104 malaria naïve adults challenged with the bites of five mosquitoes infectious with the homologous 3D7 strain of *P. falciparum*. 36 volunteers receiving RTS,S/AS01 vaccination were challenged, and 17 were completely protected from infection. 9 of those that were completely protected from infection were re-challenged 5 months later. 44 of the volunteers receiving RTS,S/AS02 vaccination were challenged, and 14 were completely protected from infection. 9 of those that were completely protected were re-challenged 5 months later. 24 of the controls were challenged at the first round; the remaining 12 were challenged 5 months later. On 85 of the occasions when vaccinated volunteers were challenged, measurements of vaccine-induced immune responses were available. Following challenge, volunteers were assessed by blood smears taken twice daily starting on day 6.5 until day 14 and then once daily until the end of the study period at day 21. Volunteers who tested positive for malaria parasites at any point in the study were then treated with chloroquine, irrespective of symptoms.

Anti-CSP antibodies were measured by evaluating IgG responses to the *P. falciparum* CSP-repeat region measured using enzyme-linked immunospot assay (ELISA). Measurements of antibody titre were analysed in units of µg/mL. In Phase II and Phase III field trials of RTS,S, antibody titres have been reported in ELISA units (EU/mL). The number of CD4^+^ or CD8^+^ T cells responding to CSP antigen and expressing the immune markers CD40L, IFN-γ, IL-2 and TNF-α per million CD4^+^ or CD8^+^ T cells were also measured (see Kester [Kester] for further details). RTS,S induced CD8^+^ T cell responses were minimal and were not associated with protection from infection. The measure of cell-mediated immunity (CMI) used in this analysis is the number of CD4^+^ T cells expressing ≥2 immune markers per million CD4^+^ T cells. Data on time to onset of parasitemia and antibody and cellular responses from control and vaccinated volunteers at both challenge and re-challenge were analyzed. Correlations between immune responses and comparisons between protected and infected volunteers are presented in the Supplementary Information in (Table S5 in File S1).

### Sporozoite Infection Model

Data from mosquito feeding studies indicates that the number of inoculated sporozoites is highly variable [31]–[33], and hence we assume the number of sporozoites inoculated during each infectious challenge follows a Negative Binomial distribution with probability mass function S(n, σ_n_ ) where the mean (*n)* and standard deviation (σ_n_) are parameters to be estimated and *S_k_* is used to denote the probability that *k* sporozoites initiate blood-stage infection. Each sporozoite that survives liver stage development is assumed to initiate blood-stage infection by releasing μ merozoites into the blood stream *t_L_* = 6.5 days after challenge [18]. We assume the number of merozoites released per sporozoite follows a Gamma distribution with mean (μ) and standard deviation (σ_μ_) estimated during model fitting. Once in the blood, parasites begin replication increasing in number by a fixed factor *m* per day [20] (Table 1) until parasite numbers reach levels *p_T_* sufficient for detection by slide microscopy [34]. Thus the duration of time between emergence of merozoites from the liver and detection (the delay in onset of parasitemia) can be used to estimate the reduction in merozoites emerging from the liver, with greater delays corresponding to greater reductions. Specifically, if *Q* denotes the number of merozoites that initiate blood-stage infection then an estimate can be obtained from the time *T* of detection as 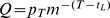
_._

**Table 1:** Parameters describing the biology of *P. falciparum* infection.

Parameter	Description	Value	Reference
***_n_***	mean number of successful sporozoites per challenge	150 (75–237)	estimated
**_σn_**	standard deviation of number of sporozoites	194 (93–324)	estimated
***t_L_***	duration of liver-stage development	6.5 days	Murphy *et al* [18]
**µ**	mean number of merozoites released per sporozoite	2,136 (1,834–3,606)	estimated
**σ_µ_**	standard deviation in number of merozoites per sporozoite	4,460 (3,394–7,613)	estimated
***_m_***	daily blood-stage parasite multiplication rate	3.8 day^−1^	Bejon *et al* [20]
***P_T_***	threshold number of parasites for detection of infection	50,000,000 parasites	Bejon *et al* [41]
**β_ab_**	anti-CSP titre for 50% reduction in sporozoite survival probability	6.62 (1.34–16.29) µg/mL	estimated
**α_ab_**	shape parameter for antibody dose-response	1.32 (0.85–1.77)	estimated
**β_CMI_**	number of CD4^+^ T cells for 50% reduction in sporozoite survival probability	1,367 (795–4,662) cells/million	estimated

Estimated parameters are shown with 95% confidence intervals.

To prevent malaria infection, we assume that all sporozoites must be killed, either by chance, by innate immune responses, or by vaccine-induced immune responses. Prevention of infection or reduction in parasite load in vaccinated volunteers following *P. falciparum* challenge is assumed to depend on anti-CSP antibody titres and the number of CSP-specific CD4^+^ T cells as well as other factors which are captured as inter-individual variation. Parametric dose-response curves (a method commonly used in the pharmacological literature [35]) are used to relate the anti-CSP titres and/or number of CSP-specific CD4^+^ T cells to the observed probability of a volunteer being protected following *P. falciparum* challenge. Exponential and Hill-functions were considered for the parametric dose response curves. For an exponential dose-response, the probability of a sporozoite surviving an immune response of magnitude *x* is 
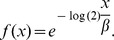
 For a Hill-function dose-response the probability is 
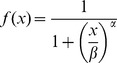
. Several markers of vaccine-induced cellular responses were available and considered alone and in combination: CSP-specific T cells expressing TNF-α, IL-2, IFN-γ or CD40L.

### Model Fitting

The model was fitted to patient data from both the vaccine and control cohorts using maximum likelihood methods. Let 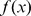
denote the probability that a single sporozoite will release merozoites from the liver given antibody levels *x_ab_* and T cell number *x_cmi_*, then the probability that *k* sporozoites from an infectious bite will release merozoites is given by 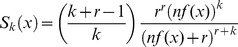 where 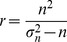 is a shape parameter of the Negative Binomial distribution. If Θ denotes the vector of parameters to be estimated and *I* indicates those protected (*I = 0*) or infected (*I = 1*) then the data likelihood given infection status *I*, merozoites emerging from the liver *Q*, and immune response *x* is:
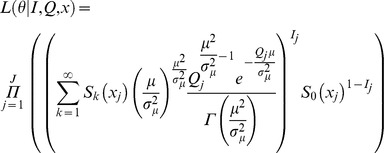
where 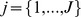 indexes the number of individuals in the study and *k* indexes the number of sporozoites injected.

The likelihood can be interpreted as follows: if volunteer 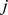 with immune marker 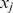 is protected (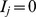) then *k = *0 sporozoites will be successful with probability 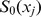. If volunteer 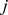 becomes infected 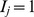, then infection will have been initiated by 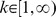 sporozoites with probability 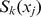. Each of the 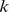
successful sporozoites will release a number of merozoites into the bloodstream following a Gamma distribution 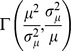. Model fits were compared using the Akaike Information Criteria (AIC). The association between anti-CSP antibodies, CSP-specific CD4^+^ T cells and protection from infection estimated by the sporozoite infection model was further validated by fitting a binary infection model where the presence or absence of infection following challenge is the only outcome of interest, and time to onset of parasitemia is ignored. Further mathematical details and likelihoods for statistical fitting, as well as simpler regression models, are given in the Supplementary Information (File S1).

### Vaccine Efficacy

Our results can be summarised in terms of two different representations of efficacy. We define efficacy against infection to be the reduction in the probability of infection following challenge from five *P. falciparum* infectious mosquitoes in vaccinated volunteers compared to control volunteers. We additionally calculate efficacy per sporozoite, defined to be the proportionate reduction in the number of sporozoites initiating blood stage infection (the liver-to-blood inoculum) [36].

### Comparative Role of the Adjuvants

To investigate the hypothesis that the higher efficacy of RTS,S/AS01 can be explained by its superior immunogenicity over RTS,S/AS02, separate dose-response curves were fitted for anti-CSP antibodies induced by RTS,S/AS01 or RTS,S/AS02.

## Results

### Sporozoite Infection Model

**Figure 1:**
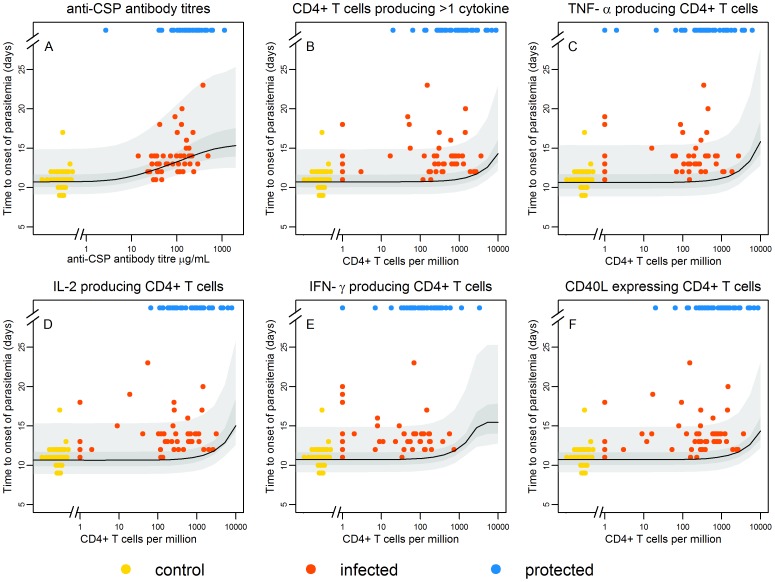
Time to onset of parasitemia. Estimated time to onset of parasitemia for those individuals that are infected as a function of anti-CSP antibody titre (A) when combined with T cells expressing two or more of TNF-α, IL-2, IFN-γ or CD40L (B), TNF-α^+^ CD4^+^ T cells (C), IL-2^+^ CD4^+^ T cells (D), IFN-γ^+^ CD4^+^ T cells (E), or CD40L^+^ CD4^+^ T cells (F). The best estimate is given by the black line and the 95% confidence intervals are shown in grey. The times to onset of parasitemia in the infectivity controls, who didn’t have detectable anti-CSP antibody titres or CSP-specific T cells are clustered on the left at (0, 8–12) (yellow points). The anti-CSP antibody titres or CSP-specific T cells of protected volunteers in whom there was no onset of parasitemia, are shown at the top for comparison (blue points). The model accurately replicates the association between time to onset of parasitemia in those that become infected (shown in gold and red) and anti-CSP antibodies (B), but does not do so for markers of cellular immunity (B–F), suggesting that the delay in parasitemia due to killing of sporozoites is best explained by anti-CSP antibody titres.

Figure 1 shows the estimated time to onset of parasitemia for a model in which sporozoite survival is dependent on anti-CSP antibody titres (A), and one of the following markers of cellular immunity: CSP-specific T cells expressing two or more of TNF-α, IL-2, IFN-γ or CD40L (B);, TNF-α^+^ CD4^+^ T cells (C);, IL-2^+^ CD4^+^ T cells (D);, IFN-γ^+^ CD4^+^ T cells (E);, and CD40L^+^ CD4^+^ T cells (F). The variation in the time to onset of parasitemia due to variation in the number of sporozoites is captured in the width of the confidence intervals. The model accurately replicates the association between time to onset of parasitemia and anti-CSP antibodies (Figure 1A), but not for markers of cellular immunity (Figure 1B–F) suggesting that the delay in parasitemia due to killing of sporozoites is predominantly attributable to antibody-mediated responses. The combination of immune markers giving the best statistical fit to the data was anti-CSP antibodies and CSP-specific CD4^+^ T cells producing two or more activation markers (see Supplementary Information, Tables S1 and S2 in File S1).

Two functional forms for the shape of the relationship between anti-CSP antibody titres or numbers of CSP-specific CD4^+^ T cells and the probability that a single sporozoite survives intra-hepatocytic development (dose-response curves) were tested. Models with interaction between the antibody- and cell-mediated responses were also tested (see Supplementary Information, Table S1 in File S1), but didn’t result in a better statistical fit to the data indicating no evidence that antibodies and cellular responses confer protection synergistically. The model providing the best fit to the data assumes that the probability of sporozoite survival decreases with increasing anti-CSP antibody titres according to a Hill function dose-response, and with increasing numbers of CSP-specific CD4^+^ T cells according to an exponential dose-response curve. The best fit parameters are shown in Table 1. We estimated the mean number of sporozoites that successfully completed intra-hepatocytic development to be 150 (95% CI 75–237) with standard deviation 194 (95% CI 93–324). We estimate that the number of merozoites released per sporozoite follows a Gamma distribution with mean 2,136 (95% CI 1,834–3,606) and standard deviation 4,460 (95% CI 3,394–7,614). The estimates of the mean and variance of the numbers of sporozoites and merozoites are dependent on the fixed parameter values in Table 1 for the duration of liver-stage development and the daily blood-stage multiplication rate (sensitivity analysis provided in Supplementary Information, Table S9 in File S1).

The model assumption that sporozoite survival is dependent on markers of antibody- and cell-mediated immunity was validated by fitting a series of nested models where these assumptions were adjusted. Table 2 shows the outcome of model fits where sporozoite survival depends on (i) anti-CSP antibody titre and numbers of CSP-specific CD4^+^ T cells; (ii) anti-CSP antibodies only; (iii) CSP-specific CD4^+^ T cells only; and (iv) vaccination status only. The large differences in the Akaike Information Criteria (AIC, a measure of model goodness-of-fit) between models indicates that there is statistical evidence that a model including both antibody and cell-mediated immune responses provides a better fit to the data than models with either alone, and that a model with antibodies alone provides a better fit to the data than a model with CSP-specific CD4^+^ T cells alone.

**Table 2:** Comparison of models where protection from infection and time to onset of parasitemia depend on (i) anti-CSP antibodies and CSP-specific CD4^+^ T cells; (ii) anti-CSP antibodies only; (iii) CSP-specific CD4^+^ T cells only; and (iv) vaccination status only.

	Parameter estimates								
Model	*n*	σ*_n_*	µ	σ_µ_	*VE*_s_	β_ab_	α_ab_	β_CMI_	ΔAIC
antibodies & CD4+ T cells	150	194	2136	4460	–	6.62	1.32	1367	0
antibodies only	156	210	2056	4205	–	5.83	1.38	–	4.34
vaccine status only	74	96	4463	9340	0.97	–	–	–	29.30
CD4^+^ T cells only	202	447	658	1444	–	–	–	489	76.62

Parameters are as defined in Table 1 and *VE_s_* is the probability that a sporozoite is killed for the vaccination status only model. The ranking of models by AIC highlights the finding that the data are best explained by a model that includes both anti-CSP antibody titres and numbers of CD4^+^ T cells, and that a model with anti-CSP antibody titres only fits better than one with CD4^+^ T cells only.

### Vaccine Efficacy Against Infection

**Figure 2:**
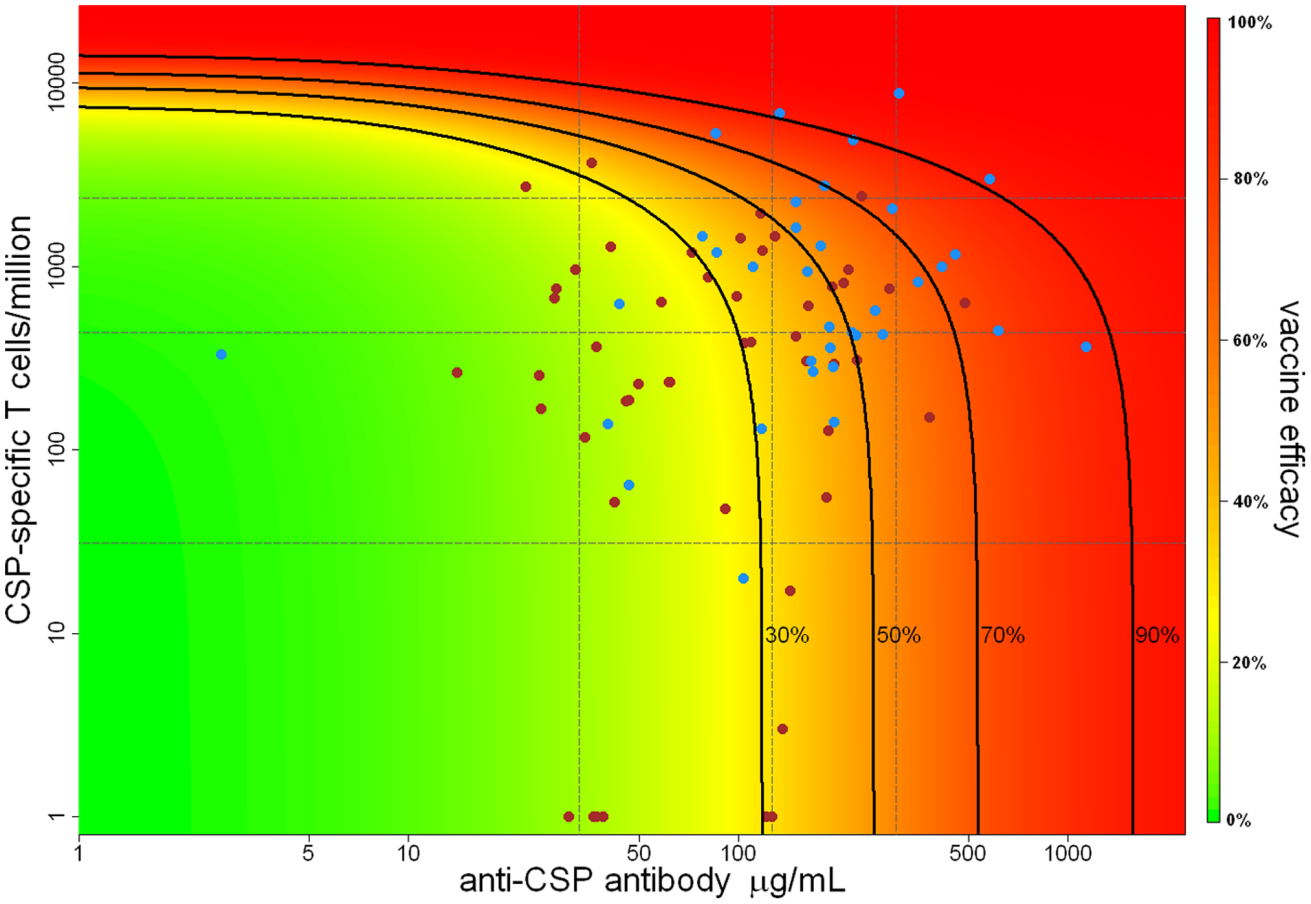
Efficacy against infection as a function of anti-CSP antibody titre and CSP-specific CD4^+^ T cells. Estimated efficacy against infection as a function of anti-CSP antibody titre and numbers of CSP-specific CD4^+^ T cells per million obtained from the sporozoite model. The vertical dashed grey lines denote the median and 90% ranges of the observed anti-CSP antibody titres, and the horizontal dashed grey lines denote the median and 90% ranges of observed numbers of CSP-specific CD4^+^ T cells. The solid black lines denote the isoclines for 30%, 50%, 70%, and 90% vaccine efficacy against infection. The blue and brown points denote the anti-CSP antibody titres and numbers of CSP-specific CD4^+^ T cells of protected and infected volunteers, respectively.

Table 3 compares the observed and model predicted efficacy against infection (defined as the prevention of blood-stage infection i.e. all sporozoites prevented from surviving intra-hepatocytic development following five bites) for the volunteers stratified into three equally sized groups by anti-CSP antibody titre and number of CSP-specific CD4^+^ T cells. Efficacy against infection in volunteers with the highest category of anti-CSP antibody titres and of CSP-specific CD4^+^ T cells is estimated to be 79% (95% CI, 58%–89%). Efficacy against infection predicted by our model as a continuous function of anti-CSP antibody titres and CSP-specific CD4^+^ T cells is shown in Figure 2. In the absence of cellular responses, vaccine-induced anti-CSP antibodies are estimated to provide 32% (95% CI, 24%–41%) protection from infection. Including the effect of the additional sporozoites killed by the CSP-specific cellular response brings the model predicted efficacy against infection to 40% (95% CI, 34%–48%). The existence of a highly protected subgroup of volunteers suggests that efficacy against infection in excess of 70% is possible if both anti-CSP antibody titres and numbers of CSP-specific CD4^+^ T cells can be boosted to high enough levels.

**Table 3:** Comparison of predicted (black) and observed (blue) efficacy against infection for the sporozoite infection model.

		anti-CSP antibody titre (µg/mL)			
		2.7–78	78–183	183–1136	0–1136
**CSP-specific CD4+ T cells**	1–268	12.6%	31.8%	47.6%	23.1%
		12.5% (2/16)	37.5% (3/8)	25% (1/4)	21.4% (6/28)
	268–820	12.6%	37.0%	57.3%	43.2%
		33.3% (2/6)	14.3% (1/7)	62.5% (10/16)	44.8% (13/29)
	820–8798	25.7%	51.9%	79.1%	54.1%
		16.7% (1/6)	64.3% (9/14)	75.0% (6/8)	57.1% (16/28)
	1–8798	15.4%	42.8%	62.1%	40.2%
		17.9% (5/28)	44.8% (13/29)	60.7% (17/28)	41.2% (35/85)

Volunteers have been stratified into low, medium and high groups according to their anti-CSP antibody titre and number of CSP-specific T cells per million.

### Vaccine Efficacy Per Sporozoite

**Figure 3:**
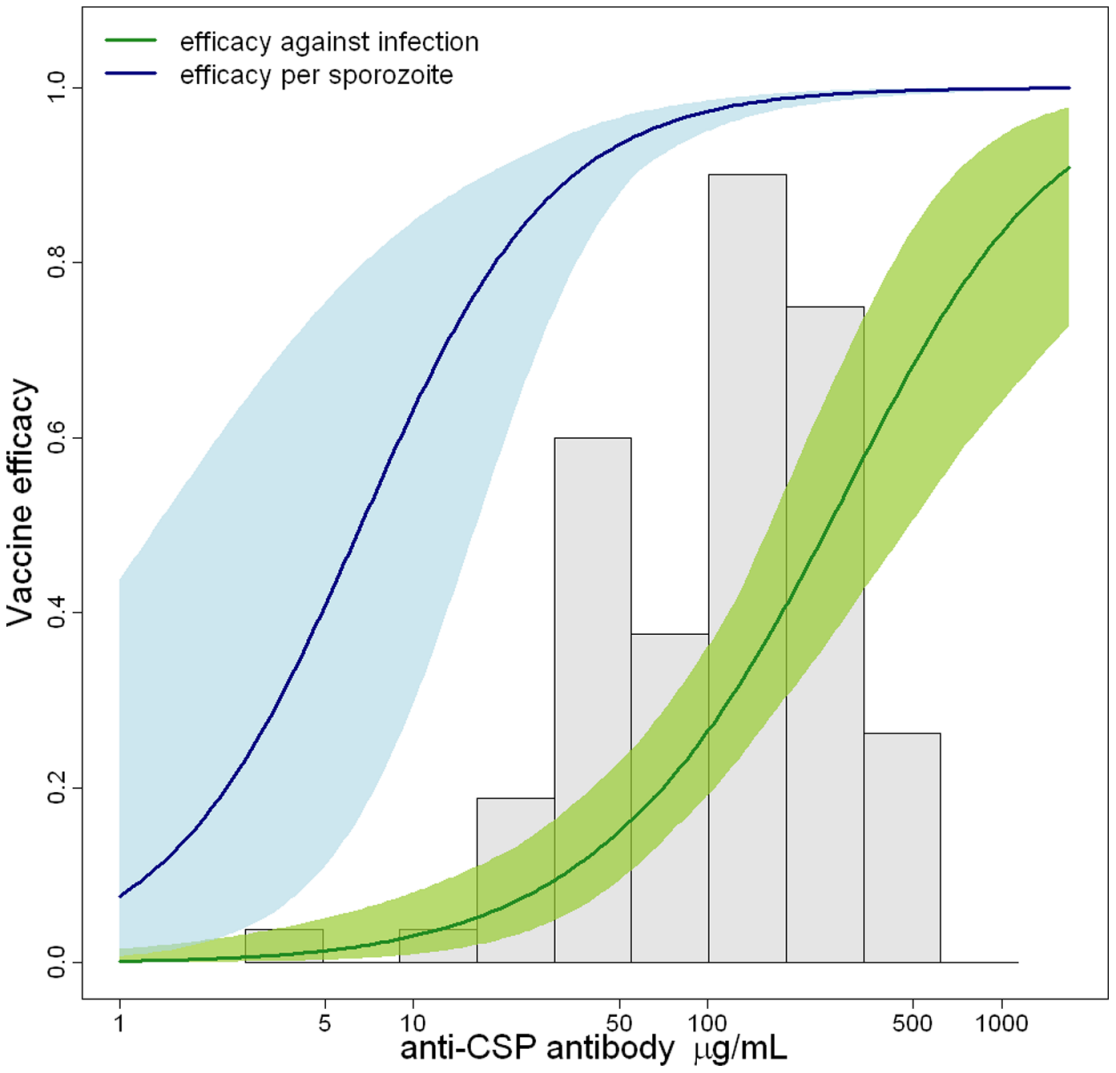
Comparison of efficacy against infection and efficacy per sporozoite. Estimated efficacy against infection (green) and efficacy per sporozoite (blue) with 95% confidence intervals as a function of anti-CSP antibody titres obtained using the sporozoite model. A histogram of the distribution of anti-CSP antibody titres is shown in grey.

We estimate the vaccine efficacy per sporozoite (defined as the percentage reduction in the number of sporozoites initiating blood stage infection – the proportion of parasites killed as opposed to the proportion of infections prevented) to be 96.1% (95% CI, 93.5%–97.8%) suggesting that a very small number of parasites are responsible for breakthrough infection. The striking discrepancy between the proportion of parasites blocked and the proportion of infections prevented is a consequence of the large number of inoculated sporozoites and the potential for a single sporozoite to initiate blood-stage infection. For volunteers vaccinated with RTS,S/AS02, we estimate efficacy per sporozoite to be 95.3% (95% CI, 92.3%–97.3%), and for those vaccinated with RTS,S/AS01 to be 97.2% (95% CI, 95.0%–98.4%). Both efficacy against infection and efficacy per sporozoite increase with increasing anti-CSP titres and numbers of CSP-specific CD4^+^ T cells (Figure 3). These estimates assume that sporozoites act independently and may be lower if this assumption does not hold.

### Effect of Adjuvant Formulation on Vaccine Efficacy

**Figure 4:**
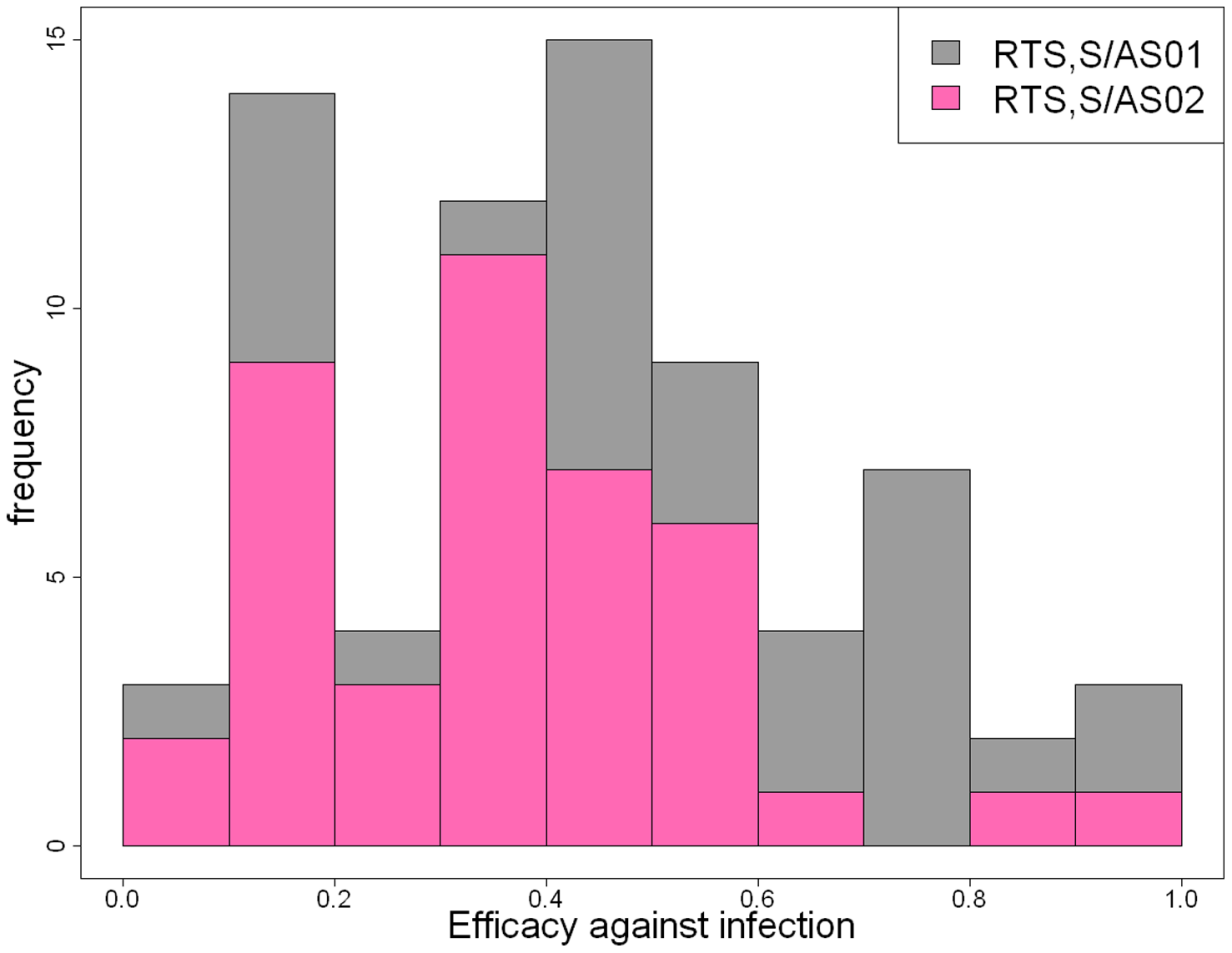
Distribution of efficacy against infection. Estimated distribution of efficacy against infection induced by both RTS,S/AS02 and RTS,S/AS01.

Vaccination is predicted to prevent infection in 40% (95% CI, 34%–48%) of challenges. For volunteers vaccinated with RTS,S/AS02, we estimate efficacy against infection to be 34% (95% CI, 28%–41%), and for those vaccinated with RTS,S/AS01 to be 48% (95% CI, 40%–57%). Although, overall, RTS,S/AS01 is more efficacious than RTS,S/AS02, there is a substantial variation in efficacy among vaccinated volunteers. Figure 4 shows the estimated distribution of efficacy against infection for both RTS,S/AS02 and RTS,S/AS01. The distribution of efficacy is consistent with RTS,S being a leaky vaccine, but with substantial variation in efficacy [37], [38].

**Figure 5:**
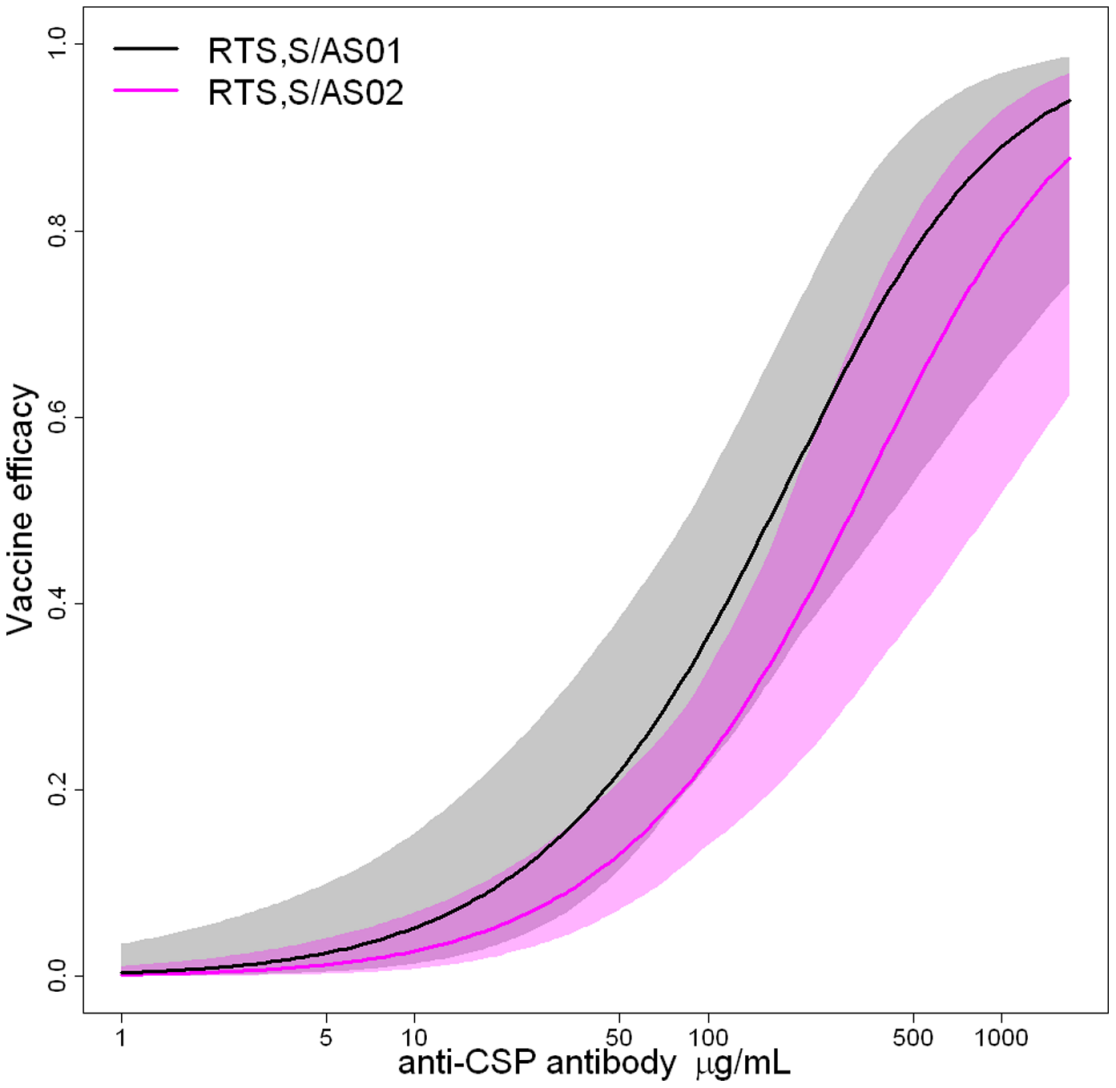
Comparison of efficacy against infection for RTS,S/AS01 and RTS,S/AS02. Comparison of efficacy against infection as a function of anti-CSP antibody titre in the absence of CSP-specific CD4^+^ T cells for RTS,S/AS01 (black) and RTS,S/AS02 (pink) based on the sporozoite infection model. The grey and pink shaded regions denote 95% confidence intervals for the estimated efficacy of RTS,S/AS01 and RTS,S/AS02, respectively. The substantial overlap between the two curves indicates that, conditional upon the magnitude of the induced antibody response, RTS,S/AS01 and RTS,S/AS02 have comparable efficacy. That is, the superior efficacy of RTS,S/AS01 over RTS,S/AS02 is estimated here to be due to the greater magnitude of the induced immune response and not some other property of the adjuvant.

A comparison of efficacy against infection as a function of anti-CSP antibody titres is shown in Figure 5 for RTS,S/AS01 and RTS,S/AS02. There was no significant difference in the relationship between anti-CSP titres and efficacy against infection, indicating that adjuvant formulation does not alter the quality of the induced immune responses, but contributes to protection only by increasing the magnitude of induced immune responses. This is equivalent to the result that anti-CSP antibody titres but not adjuvant formulation satisfy the Prentice criterion [39] to be a surrogate of protection which can be demonstrated using logistic regression models (see Supplementary Information, Table S8 in File S1). The Prentice surrogate definition is not suitable for comparison between vaccinated and control volunteers as there is not substantial variability in immune responses in the control volunteers [40], although the data are consistent with anti-CSP antibodies and numbers of CSP-specific T cells being surrogates of protection as defined by Qin *et al* [40].

## Discussion

Using an individual level within-host mathematical model of the processes underlying sporozoite infection, we were able to extend upon Kester *et* al’s [29] analysis of aggregated data on immune responses and time to onset of parasitemia to provide evidence to support a number of hypotheses for the mechanism of action of the RTS,S vaccine: (i) anti-CSP antibody titres and CSP-specific CD4^+^ T cells constitute surrogates of protection against infection in the absence of exposure-driven confounding; (ii) when adjusted for vaccine-induced antibody titres and numbers of CSP-specific CD4^+^ T cells, there is no dependence of vaccine efficacy on adjuvant formulation, i.e. the adjuvant increases the magnitude of the response but does not change its nature; (iii) RTS,S-induced immune responses kill greater than 95% of sporozoites suggesting that in infected volunteers, the liver-to-blood inoculum may often be the progeny of a single sporozoite; and (iv) RTS,S is a leaky vaccine with substantial heterogeneity between individuals in vaccine efficacy against infection.

Our model results predict that RTS,S-induced protection from infection is dependent on both anti-CSP antibodies and CSP-specific CD4^+^ T cells, with antibodies playing a dominant role in preventing infection. However, this finding is based on the number of CSP-specific CD4^+^ T cells per million expressing ≥2 of the cytokines IL-2, TNF-α, IFN-γ or the co-stimulatory molecule CD40L as a marker of cellular immunity [29]. We did not find any other combination of these parameters to be more predictive, in contrast to other studies suggesting that CSP-specific CD4^+^ T cells producing TNF-α have been associated with a reduced risk of clinical malaria in a trial of RTS,S/AS01 in children [25]. Other sub-populations of T cells may be more strongly associated with vaccine efficacy with Lumsden *et al* observing an association between IL-2 and TNF-α producing effector and central memory CD4^+^ T cells and protection [41]. A possible hypothesis for the higher level of protection conferred by antibodies than cellular responses is the random nature in which a T cell encounters an infected hepatocyte. Furthermore, RTS,S-induced T cell responses are relatively low compared with classic T cell inducing vaccines such as BCG, whereas the antibody response is high compared to most other vaccines.

RTS,S has been observed to be more immunogenic when formulated with AS01 compared to when formulated with AS02 [13]. This was demonstrated in the original study reporting these data which summarized a range of immune markers in the two groups. In endemic settings, RTS,S/AS02 has been observed to have 30% (95% CI, 11%–45%) efficacy against first episodes of clinical malaria in Mozambican children [7], compared to RTS,S/AS01 which was observed to have 53% (95% CI, 28%–69%) efficacy against first episodes in Kenyan and Tanzanian children [8]. Our results demonstrate that the differences in observed efficacy for these two formulations of RTS,S can be explained by the induction of higher anti-CSP antibody titres and greater numbers of CSP-specific CD4^+^ T cells by RTS,S/AS01 compared to RTS,S/AS02.

Our results indicate that RTS,S prevents the majority of *P. falciparum* parasites from surviving the pre-erythrocytic stage of infection, with an estimated efficacy per sporozoite of 96.1% (95% CI, 93.4%–97.8%). A comparably high level of efficacy per sporozoite for pre-erythrocytic vaccines has been suggested by the results of longitudinal PCR studies on challenged volunteers [20], [36]. Such high levels of efficacy per sporozoite are needed to obtain significant rates of sterile protection for individual volunteers, given that a single sporozoite evading the vaccine-induced immune response can lead to blood-stage infection. The need for such high levels of efficacy per sporozoite to result in even a partially effective vaccine is a major challenge in developing highly efficacious pre-erythrocytic malaria vaccines. No definitive threshold for protection, in terms of either anti-CSP antibody titres or CD4^+^ T cell responses, was identified by our model. Instead, vaccine efficacy was estimated to increase monotonically, albeit non-linearly, across the range of observed antibody titres and cellular responses. With the reduced liver-to-blood inocula (often the progeny of a single sporozoite) due to vaccination with RTS,S, there is the possibility that a combination vaccine that also induces immune responses against blood-stage parasites could eliminate the few merozoites that do emerge from the liver, although this was not evident in a study evaluating RTS,S in combination with MSP-1 [42].

There was significant variation between volunteers in the anti-CSP antibody titres and numbers of CSP-specific CD4^+^ T cells on the day of challenge. Under the sporozoite infection model, this variation in response predicts substantial between-person heterogeneity in vaccine efficacy; that is, partial protection is induced in everyone but some individuals are more protected than others. Thus the vaccine is estimated to be leaky but with substantial variation in efficacy between volunteers. However the findings presented here only apply on the day of challenge or re-challenge and do not give information on the duration of protection. The nature of infectious challenge with malaria may also contribute to variation in vaccine efficacy as infections arising from bites with a small number of inoculated sporozoites may be easily prevented, whereas infections arising from large doses of sporozoites may be difficult to prevent.

There are a number of limitations to the sporozoite infection model which describes a simplified version of the processes underlying *P. falciparum* infection. Firstly, as a mosquito inoculates sporozoites into the skin, the probability that one sporozoite evades the vaccine-induced immune response may not be independent of the survival of other sporozoites. For example, if the inoculation site is near a blood vessel then all sporozoites may evade vaccine-induced immune responses and migrate to the liver in the blood stream. If the probabilities of sporozoite survival are not independent, then the efficacy per sporozoite of the vaccine may be substantially lower than our estimate. Secondly, variation in both the number of sporozoites and the number of merozoites per sporozoite will contribute to variation in the liver-to-blood inocula. For example, high variance in liver-to-blood inocula could be a result of a constant number of sporozoites releasing a variable number of merozoites, or a variable number of sporozoites each releasing a constant number of merozoites. Furthermore, we have assumed a constant growth rate for blood-stage parasites. Variation between individuals in this growth rate may additionally contribute to the variation in time to detection of parasites. The magnitude of the induced immune responses and the association between CD4^+^ T cells and protection from infection may depend on genetic variation between volunteers, particularly variation in class II MHC expression which is responsible for priming CD4^+^ T cells [43].

Our results suggest that the RTS,S vaccine acts through the induction of high levels of both anti-CSP antibodies and CSP-specific CD4^+^ T cells, with the antibody response having a greater role. These results can potentially be utilised in endemic settings by identifying individuals receiving RTS,S who have generated low vaccine-induced antibody responses and are therefore at greater risk of re-infection, and who may become a priority for receiving a booster dose. Identifying covariates such as age, exposure to malaria and malnutrition that are associated with the magnitude of vaccine-induced immune responses in children in endemic areas will aid in the evaluation of the impact of programmes of wide scale vaccination with RTS,S. Furthermore, the existence of a subgroup of volunteers with high antibody and cell-mediated immune responses who display an estimated vaccine efficacy against infection greater than 70% suggests that substantial increases in efficacy can be obtained if vaccine immunogenicity can be further improved.

## Supporting Information

File S1 (DOCX)
