# Supplementary material for: The Relationship between RTS,S Vaccine-Induced Antibodies, CD4+ T Cell Responses and Protection against Plasmodium falciparum Infection
Source: PLoS One. 2013 Apr 16;8(4):e61395. doi: 10.1371/journal.pone.0061395 (PMC3628884; doi:10.1371/journal.pone.0061395)
Supplement: File S1 — (DOCX) [file pone.0061395.s001.docx]

**The relationship between RTS,S vaccine-induced antibodies, CD4^+^ T cell responses and protection against *Plasmodium falciparum* infection**

*Supplementary Information*

Michael T. White^1*^, Philip Bejon^2,3^, Ally Olotu^2^, Jamie T. Griffin^1^, Eleanor M. Riley^4^, Kent E. Kester^5^, Christian F. Ockenhouse^5^, Azra C. Ghani^1^

1. MRC Centre for Outbreak Analysis and Modelling, Department of Infectious Disease Epidemiology, Imperial College London
2. Kenya Medical Research Institute, Kilifi, Kenya
3. Centre for Clinical Vaccinology and Tropical Medicine, University of Oxford, UK
4. Department of Immunology and Infection, Faculty of Infectious and Tropical Diseases, London School of Hygiene and Tropical Medicine
5. Walter Reed Army Institute of Research, Silver Spring, Maryland

*Address for correspondence: Dr Michael White, Department of Infectious Disease Epidemiology, Imperial College London, Norfolk Place, London W2 1PG. Email: [m.white08@imperial.ac.uk](mailto:m.white08@imperial.ac.uk)

**Model methods**

**Sporozoite infection model**

Malaria-naïve volunteers were challenged with the bites of five *P. falciparum* infectious mosquites. Data from mosquito feeding studies indicates that the number of inoculated sporozoites is highly variable [[1-3](#_ENREF_1)]. We assume that the number of sporozoites inoculated into the skin by five mosquitoes follows a Negative Binomial distribution. If a fixed proportion of these successfully complete hepatic development, then the number of sporozoites initiating blood-stage infection will be Negative Binomially distributed with mean *n* and variance $\sigma_{n}^{2}=\frac{n(n+r)}{r}$, where *r* is a shape parameter . The probability that after challenge, *k* sporozoites will initiate blood-stage infection is then

$$S_{k}=\left(\begin{matrix} k+r-1 \\ k \end{matrix}\right)\frac{r^{r}n^{k}}{\left(n+r\right)^{r+k}}$$

Each sporozoite that survives liver stage development will initiate blood-stage infection by releasing merozoites into the blood stream after *t_L_  =* 6.5 days [[4](#_ENREF_4)]. We assume the number of merozoites is Gamma distributed with mean *µ* and standard deviation σ_µ_. Once in the blood, merozoites will begin replication increasing in number by a factor *m* = 3.8 per day [[5](#_ENREF_5)]. Periodic fluctuations in parasite density due to sequestration, short term changes in innate immunity, and inter-individual differences in parasite replication rates are ignored. If blood-stage infection is initiated with *Q* merozoites, then the number of parasites at time *t* is

$$P(t)=\left\{\begin{matrix} 0 t<t_{L} \\ Qm^{t-t_{L}} t\geq t_{L} \end{matrix}\right.$$

Blood-stage infection can be diagnosed once parasite density reaches a threshold of 10,000 parasites/mL [[6](#_ENREF_6)], corresponding to a total of *p_T_*=50,000,000 parasites. If onset of parasitemia is observed on day *T*, the initial infectious dose of merozoites can be estimated as$Q=p_{T}m^{-(T-t_{L})}$. Thus the delay in onset of parasitemia can be used to estimate the reduction in merozoites emerging from the liver, with greater delays corresponding to greater reductions.

**Dose-response curves**

Vaccine-induced immune responses can prevent infection or reduce parasite load by killing *P. falciparum* sporozoites in the skin or liver [[7](#_ENREF_7)]. The proportion of sporozoites killed by vaccine-induced immune responses is likely to depend on anti-CSP antibody titres and numbers of CSP-specific CD4^+^ T cells [[8](#_ENREF_8)] as well as other factors such as innate immune responses and naturally acquired pre-erythrocytic immunity. Although it may not be possible to precisely characterise the relationship between immune markers and protection from infection, an approximate relationship can be estimated using a dose-response curve [[9](#_ENREF_9)].

Given a dose *x* (e.g. anti-CSP antibody titre, CSP-specific CD4^+^ T cells) the response *f* (probability of sporozoite success) can be estimated. The following dose-response curves were considered:

1. Exponential $f(x)=e^{-\log(2)\frac{x}{\beta}}$
2. Hill function $f(x)=\frac{1}{1+\left(\frac{x}{\beta}\right)^{\alpha}}$

where $\alpha$ is a shape parameter and$\beta$ is a scale parameter. The response may depend on the dose of two different quantities, say anti-CSP antibody titre *x_ab_*, and number of CSP-specific CD4^+^ T cells *x_cmi_*. If these immune mechanisms provide protection independently of each other then their dose-response curves can simply be multiplied to give

$$f(x_{\text{ab}},x_{\text{cmi}})=f(x_{\text{ab}})f(x_{\text{cmi}})$$

There may be synergistic interactions between vaccine-induced antibody-mediated and cell-mediated immunity, making a combination of anti-CSP antibodies and CSP-specific CD4^+^ T cells more efficacious than the combined effect of each immune component acting in isolation [[10](#_ENREF_10)]. Alternatively, antibody-mediated and cell-mediated responses may have a less than multiplicative effect when combined. For example, if an antibody response prevents 50% of sporozoites and a cellular response also prevents 50% of sporozoites, then a combined response may only prevent 60% of sporozoites, in contrast to the 75% that would be expected if the responses were multiplicative. Following the approaches of Greco *et al* [[11](#_ENREF_11)] and White *et al* [[12](#_ENREF_12)] interaction between immune responses can be modelled using the following dose-response function

$$f(x_{\text{ab}},x_{\text{cmi}})=e^{-\frac{\log(2)}{2}\left(\left(\frac{x_{\text{ab}}}{\beta_{\text{ab}}}+\frac{x_{\text{cmi}}}{\beta_{\text{cmi}}}\right)+\sqrt{\left(\frac{x_{\text{ab}}}{\beta_{\text{ab}}}+\frac{x_{\text{cmi}}}{\beta_{\text{cmi}}}\right)^{2}+4\gamma\frac{x_{\text{ab}}x_{\text{cmi}}}{\beta_{\text{ab}}\beta_{\text{cmi}}}}\right)}$$

where$\gamma$ is a shape parameter, and$\beta_{\text{ab}}$, $\beta_{\text{cmi}}$are scale parameters. Here$\gamma>0$results in a dose-response surface with synergistic interaction, $\gamma=0$results in a dose-response surface where each immune component acts independently, and $-1<\gamma<0$results in a less than multiplicative combination of immune responses.

If $x=\left(x_{\text{ab}},x_{\text{cmi}}\right)$ is the vector of immune markers anti-CSP antibody titres and number of CSP-specific CD4^+^ T cells, then the mean number of successful sporozoites will be reduced from *n* to *nf*(*x*). The probability that *k* sporozoites are inoculated in the presence of immune response *x* is then given by

$$S_{k}\left(x\right)=\left(\begin{matrix} k+r-1 \\ k \end{matrix}\right)\frac{r^{r}\left(nf(x)\right)^{k}}{\left(nf(x)+r\right)^{r+k}}$$

**Model likelihood**

Under the sporozoite infection model, the probability of infection and the expected time to onset of parasitemia will depend on the mean and standard deviation of the number of successful sporozoites (*n*,σ*_n_*), the mean and standard deviation of the number of merozoites released per sporozoite (µ,σ_µ_) and the parameters describing the dose-response relationship between anti-CSP antibody titre, numbers of CSP-specific CD4^+^ T cells and the probability of sporozoite infection (β_ab_,α_ab_, β_cmi_,α_cmi_). Denote Ѳ to be the vector of parameters to be estimated, $I$an indicator for protected or infected, and *T* the time to onset of parasitemia, then the likelihood is given by

where$j=\{1,...,J\}$indexes the number of individuals in the study and *k* indexes the number of sporozoites injected.

The likelihood in equation (8) can be interpreted as follows: if volunteer$j$with immune marker$x_{j}$is protected ($I_{j}=0$) then 0 sporozoites will be successful with probability$S_{0}(x_{j})$. If volunteer$j$becomes infected$I_{j}=1$, then infection will have been initiated by $k\in[1,\infty)$sporozoites with probability$S_{k}(x_{j})$. Each of the$k$successful sporozoites will release a number of merozoites into the bloodstream following a Gamma distribution.

**Time to onset of parasitemia**

Given a model estimate of the liver-to-blood inocula $Q(x)$in a volunteer with vaccine-induced immune response *x*, the time to onset of parasitemia following *P. falciparum* challenge can be estimated as

Under the sporozoite infection model, blood-stage infection must be initiated by at least one sporozoite releasing merozoites into the blood stream. The mean and variance of the time to onset of parasitemia can be simulated by estimating the number of merozoites released from the liver$Q(x)$. This is done by simulating the number of successful sporozoites (Negative Binomial distribution) and then the number of merozoites released by the sporozoites (Gamma distribution). The sporozoite infection model does not capture delays in onset of parasitemia due to inter-individual variation in blood-stage parasite replication rates [[5](#_ENREF_5),[13](#_ENREF_13),[14](#_ENREF_14)] and variation in the density at which parasites become detectable by slide microscopy [[6](#_ENREF_6)].

**Efficacy against infection and efficacy per sporozoite**

Given immune response *x*, the efficacy per sporozoite *VE_s_* can be estimated as the proportional reduction in the number of parasites emerging from the liver

$$VE_{s}(x)=1-\frac{n\mu f(x)}{n\mu}=1-f(x)$$

Infection can be prevented outright if all sporozoites are killed, i.e. if the number of successful sporozoites is zero. We define efficacy against infection *VE_i_*  to be the reduction in the probability of infection of vaccinated compared to control volunteers. As the number of successful sporozoites is assumed to follow a Negative Binomial distribution, the efficacy against infection can be estimated as

**Binary infection model**

The sporozoite infection model utilises the data available in the binary outcome of infection or protection, and the data in time to onset of parasitemia in infected volunteers. An alternative approach is to ignore the reduction in parasite load and treat infection as a purely binary outcome. After *P. falciparum* challenge a volunteer will either become infected$I=1$or remain uninfected$I=0$depending on their anti-CSP antibody titre and number of CSP-specific CD4^+^ T cells *x*. The likelihood that the parameters$\theta=\{\alpha,\beta\}$of a dose-response curve *f* generate the observed data can be written as

$$L\left(\theta\left|I,x\right.\right)=\prod_{j=1}^{J} f(x_{j})^{I_{j}}\left(1-f(x_{j})\right)^{1-I_{j}}$$

The results of fitting the binary infection model to the data are shown in Table S3 and Table S4.

**Comparative role of the adjuvants**

To investigate the hypothesis that the higher efficacy of RTS,S/AS01 can be explained by its superior immunogenicity over RTS,S/AS02, the data from vaccinated volunteers was split by adjuvant formulation: AS01 or AS02. The binary infection and sporozoite models were then fitted separately to each data set.

**Calculation of confidence intervals**

The best fit parameters were estimated as the parameter vector$\hat{\theta}$that maximises the likelihood$L\left(\left.\theta\right| \text{ }\right)$. The set of parameters$\theta_{95}$that fall within the 95% confidence intervals were calculated as those parameters which could not be rejected when compared to the maximum likelihood parameters $\hat{\theta}$using the likelihood ratio test

$$\left\{\theta_{95}:2\left(\log(\hat{\theta})-\log(\theta_{95})\right)\leq\chi_{1}^{2}\left(0.05\right)=3.84\right\}$$

**Extended model fitting results**

**Sporozoite infection model**

For the sporozoite infection model, combinations of Exponential and Hill function dose-response curves for the relationship between anti-CSP antibody titres, CSP-specific CD4^+^ T cells and the probability of killing a sporozoite were tested. In addition, a model where the effects of anti-CSP antibodies and CSP-specific CD4^+^ T cells follow dose-response curves but with an interaction term was tested. A comparison of five models with different combinations of dose-response curves is presented in Table S1. The parameters were estimated by maximum likelihood (ML) methods. Models were compared and ranked for goodnes of fit using the Akaike information criterion (AIC).

Several markers of vaccine-induced cellular responses were available numbers of CSP-specific CD4^+^ or CD8^+^ T cells per million expressing the immune markers IFN-γ, IL-2, TNF-α, CD40L, or two or more of these [[15](#_ENREF_15)]. For each of the combinations of anti-CSP antibodies with one of the markers of CD4^+^ cellular immunity, the sporozoite infection model was fitted using maximum likelihood methods with the results presented in Table S2. There was no association between markers of CD8^+^ cellular immunity and protection from infection, or delay in time to onset of parasitemia.

Table S1: Maximum likelihood parameter estimates for sporozoite infection models with different combinations of dose-response curves (ab, cmi) and model comparison with Akaike information criterion (AIC). *hill-exp denotes a Hill function dose-response function for antibodies and an exponential dose-response for cellular responses. ^+^exp-exp-int denotes exponential dose-response functions for both antibody and cellular responses with an interaction term.

|  | *n* | σ_n_ | µ | σ_µ_ | β_ab_ | α_ab_ | β_cmi_ | α_cmi_ | γ | ML | AIC | ΔAIC |
| --- | --- | --- | --- | --- | --- | --- | --- | --- | --- | --- | --- | --- |
| hill-exp* | 150.4 | 194.3 | 2136 | 4460 | 6.62 | 1.32 | 1367 | – | – | -1023.39 | 2060.77 | 0 |
| hill-hill | 148.9 | 191.2 | 2155 | 4465 | 7.69 | 1.28 | 532 | 0.83 | – | -1023.58 | 2063.17 | 2.39 |
| exp-hill | 145.3 | 194.1 | 2058 | 4312 | 66.90 | – | 1.01 | 0.41 | – | -1026.65 | 2067.31 | 6.53 |
| exp-exp | 99.7 | 145.3 | 2042 | 4503 | 38.57 | – | 738 | – | – | -1038.04 | 2088.08 | 27.31 |
| exp-exp-int^+^ | 99.8 | 144.1 | 2050 | 4558 | 42.21 | – | 1064 | – | 1.90 | -1037.74 | 2089.48 | 28.71 |

Table S2: Maximum likelihood parameter estimates for sporozoite infection models with different markers of vaccine-induced cellular immune responses, and model comparison with Akaike information criterion (AIC).

|  | *n* | σ_n_ | µ | σ_µ_ | β_ab_ | α_ab_ | β_cmi_ | ML | AIC | ΔAIC |
| --- | --- | --- | --- | --- | --- | --- | --- | --- | --- | --- |
| IgG – IL2 | 151.2 | 195.1 | 2125 | 4428 | 6.85 | 1.33 | 1175 | -1023.08 | 2060.16 | 0 |
| IgG – ≥2 markers | 150.4 | 194.3 | 2136 | 4460 | 6.62 | 1.32 | 1367 | -1023.39 | 2060.77 | 0.61 |
| IgG – CD40L | 150.4 | 194.2 | 2136 | 4463 | 6.65 | 1.32 | 1361 | -1023.40 | 2060.80 | 0.64 |
| IgG – TNF-α | 148.9 | 192.7 | 2158 | 4525 | 6.08 | 1.30 | 948 | -1023.99 | 2061.98 | 1.82 |
| IgG – IFN-γ | 146.3 | 189.9 | 2197 | 4585 | 5.78 | 1.31 | 325 | -1025.36 | 2064.73 | 4.57 |

**Binary infection model**

In the binary infection model we assume a dose-response between anti-CSP antibodies, CSP-specific CD4^+^ T cells and protection from infection (not the probability of killing a single sporozoite). For the same combination of dose-response curves explored in the sporozoite infction model, the results of model fitting using maximum likelihood methods are presented in Table S3. For each of the combinations of anti-CSP antibodies with one of the markers of CD4^+^ cellular immunity, the binary infection model was fitted using maximum likelihood methods with the results presented in Table S4.

Table S3: Maximum likelihood parameter estimates for binary infection models with different combinations of dose-response curves (ab, cmi) and model comparison with Akaike information criterion (AIC).

|  | β_ab_ | α_ab_ | β_cmi_ | α_cmi_ | γ | ML | AIC | ΔAIC |
| --- | --- | --- | --- | --- | --- | --- | --- | --- |
| exp-exp | 253.9 | – | 3253 | – |  | -48.54 | 101.09 | 0 |
| exp-exp-int | 356.0 | – | 9301 | – | 11.42 | -48.46 | 102.93 | 1.84 |
| hill-exp | 240.7 | 1.34 | 3168 | – | – | -48.58 | 103.16 | 2.07 |
| exp-hill | 279.1 | – | 5164 | 0.69 | – | -48.59 | 103.19 | 2.10 |
| hill-hill | 268.8 | 1.74 | 6584 | 0.49 | – | -48.34 | 104.70 | 3.61 |

Table S4: Maximum likelihood parameter estimates for binary infection models with different markers of vaccine-induced cellular immune responses, and model comparison with Akaike information criterion (AIC).

|  | β_ab_ | β_cmi_ | ML | AIC | ΔAIC |
| --- | --- | --- | --- | --- | --- |
| IgG – TNF-α | 250.1 | 1863 | -48.15 | 100.30 | 0 |
| IgG – IL2 | 257.0 | 2798 | -48.45 | 100.89 | 0.58 |
| IgG – CD40L | 254.3 | 3218 | -48.53 | 101.07 | 0.77 |
| IgG – ≥2 markers | 253.9 | 3235 | -48.54 | 101.09 | 0.79 |
| IgG – IFN-γ | 225.4 | 625 | -50.50 | 105.01 | 4.71 |

The efficacy against infection estimated by the binary infection model is *VE_i_* = 40% (95% CI, 31% - 49%). As the binary infection model estimates the proportion of infections prevented outright, and not the proportion of sporozoites killed, it can not be used to estimate vaccine efficacy per sporozoite.

In the sporozoite infection model it is assumed that following an infectious challenge in a vaccinated volunteer, the survival of each sporozoite is independent of the survival of all other sporozoites. If instead the survival of one sporozoite is not independent of other sporozoites, we would expect to see more cases where zero or all sporozoites are killed by vaccine-induced immune responses. In the extreme case, all sporozoites would be killed in protected volunteers and no sporozoites would be killed in infected volunteers, so that the proportion of sporozoites killed equals the proportion of infections prevented, 40%.

**Two adjuvant models**

To investigate the dependence of adjuvant formulation (AS01 or AS02) on vaccine efficacy, we fitted separate dose-response curves for RTS,S/AS01 and RTS,S/AS02 induced immune responses. For RTS,S/AS01, the sporozoite infection model estimates that 50% of sporozoites are killed by a vaccine-induced anti-CSP antibody titre of β_ab_ = 3.2 (95% CI, 0.4 – 10.3) µg/mL, whereas for RTS,S/AS02 it is estimated that β_ab_ = 6.4 (95% CI, 1.4 – 14.6) µg/mL. Similarly it is estimated that 50% of sporozoites are killed by β_cmi_ = 3,796 (95% CI, 66 – 7,536) RTS,S/AS01-induced CSP-specific T cells per million, or β_cmi_ = 1,665 (95% CI, 50 – 5,304) RTS,S/AS02-induced CSP-specific T cells per million. Figure 5 shows a comparison of the dose-response curves for RTS,S/AS01 and RTS,S/AS02. The substantial overlap between the two curves indicates that conditional upon the magnitude of the induced immune responses RTS,S/AS01 and RTS,S/AS02 have comparable efficacy, that is, the superior efficacy of RTS,S/AS01 over RTS,S/AS02 is due to the greater magnitude of the induced immune response and not some other property of the adjuvant. This hypothesis is further explored using logistic regression models in the next section.

**Statistical regression models**

To complement the biologically-motivated mathematical models of the association between vaccine-induced immune responses and protection from infection previously described, we present the results of simpler statistical regression models. The magnitudes of the different vaccine-induced immune responses are correlated as demonstrated in Table S5.

Table S5: Spearman rank correlation between anti-CSP IgG antibody titre and markers of CD4^+^ CSP-specific cellular immunity.

|  | correlation | P value |
| --- | --- | --- |
| IgG – ≥2 markers | 0.259 | 0.017* |
| IgG – IFN-γ | 0.347 | 0.001* |
| IgG – IL2 | 0.206 | 0.058 |
| IgG – TNF-α | 0.265 | 0.014* |
| IgG – CD40L | 0.248 | 0.021* |

The association between RTS,S-induced anti-CSP antibodies, number of CSP-specific CD4^+^ T cells, and protection from infection or delay in onset of parasitemia can be demonstrated using statistical regression models. Table S6 shows the results of fitting linear regression models to the time of onset of parasitemia in infected volunteers. In all models, anti-CSP antibody titres but not numbers of CSP-specific CD4^+^ T cells were significantly associated with time to onset of parasitemia.

Table S6: Linear regression models of time to onset of parasitemia (T) against anti-CSP antibody titre (IgG), number of CSP-specific CD4^+^ T cells per million (CMI) and adjuvant formulation.

|  | estimate | P value |
| --- | --- | --- |
| *T ~ log(IgG) + log (CMI)* | | |
| intercept | 9.18 | 4.47 x 10^-6^* |
| log(IgG) | 1.12 | 0.005* |
| log(CMI) | -0.03 | 0.811 |
|  |  |  |
| *T ~ log(IgG) + log (CMI) + adjuvant* | | |
| intercept | 10.52 | 1.28 x 10^-6^* |
| log(IgG) | 1.03 | 0.009* |
| log(CMI) | -0.07 | 0.618 |
| adjuvant | -1.16 | 0.083 |
|  |  |  |
| *T ~ log(IgG) + log (CMI) + log(IgG)*log(CMI)* | | |
| intercept | 2.21 | 0.647 |
| log(IgG) | 2.79 | 0.018* |
| log(CMI) | 1.22 | 0.143 |
| log(IgG)*log(CMI) | -0.30 | 0.128 |

Table S7 shows the results of fitting logistic regression models to the outcome of infectious challenges. Both anti-CSP antibody titres and numbers of CSP-specific CD4^+^ T cells were significantly associated with increased protection from infection, except in the model with an interaction term. The interaction model indicates a synergistic reaction between antibodies and T cells, however it also makes the biologically unrealistic prediction that in the absence of T cells, anti-CSP antibodies are associated with increased probability of infection. In addition the interaction model had a higher AIC than the model without interaction (AIC 103.79 vs. 103.52).

Although straightforward statistical regression models can capture the association between vaccine-induced markers and protection from infection or delayed time to onset of parasitemia as demonstrated above, they cannot capture both trends in the one model. In addition they do not incorporate any understanding of the biological processes underlying *P. falciparum* infection, and so can make biologically unrealistic predictions.

Table S7: Logistic regression models of protection from infectious challenge, against anti-CSP antibody titre (IgG), number of CSP-specific CD4^+^ T cells per million (CMI) and adjuvant formulation.

|  | estimate | P value |
| --- | --- | --- |
| *protection ~ log(IgG) + log (CMI)* | | |
| intercept | -6.11 | 0.0003* |
| log(IgG) | 0.75 | 0.0117* |
| log(CMI) | 0.36 | 0.0311* |
|  |  |  |
| *protection ~ log(IgG) + log (CMI) + log(IgG)*log(CMI)* | | |
| intercept | 1.54 | 0.78 |
| log(IgG) | -0.98 | 0.45 |
| log(CMI) | -0.89 | 0.33 |
| log(IgG)*log(CMI) | 0.28 | 0.18 |

Logistic regression models can be used to test whether an immune marker satisfies the Prentice criterion to be a surrogate of protection [[16](#_ENREF_16)]. The Prentice criterion can be stated as follows

P1. Vaccination affects outcome (infection or protection).
P2. Vaccination affects surrogates (anti-CSP antibodies or numbers of CSP-specific CD4^+^ T cells).
P3. Surrogates affect outcome.
P4. Conditional upon the surrogates, outcomes are independent of vaccination status.

In particular, we focus on volunteers receiving RTS,S/AS02 or RTS,S/AS01, and show that vaccine-induced anti-CSP antibody titres and numbers of CSP-specific CD4^+^ T cells satisfy the Prentice criterion. Conditions P1, P2, and P3 have been repeatedly demonstrated to be satisfied[[15](#_ENREF_15)]. For volunteers receiving either RTS,S/AS02 or RTS,S/AS01, condition P4 is tested using a logistic regression model for the dependence of protection following infectious challenge on anti-CSP antibody titres, numbers of CSP-specific T cells and adjuvant. The results in Table S8 indicate that outcome is dependent on both anti-CSP antibody titres and numbers of CSP-specific T cells but not adjuvant, confirming condition P4.

The Prentice surrogate definition is not as useful for comparison between vaccinated and control volunteers, as there will be no variability in the surrogate marker in the control volunteers [[17](#_ENREF_17)]. Nevertheless, it can still be tested whether or not anti-CSP antibody titres or numbers of CSP-specific CD4^+^ T cells constitute principal surrogates of protection as defined by Qin *et al* [[17](#_ENREF_17)]. Control volunteers are assumed to have the lowest possible measurable immune response. The results of a logistic regression model for the effects of anti-CSP antibody titres, numbers of CSP-specific CD4^+^ T cells and vaccination status on control and vaccinated volunteers are presented in Table S8. The outcome of following infectious challenge depends significantly on both antibody and cellular responses but not vaccination status, indicating that anti-CSP antibody titres and numbers of CSP-specific CD4^+^ T cells are surrogates of protection.

Table S8: Testing for Prentice criterion or surrogate of protection using logistic regression models of protection from infectious challenge, against anti-CSP antibody titre (IgG), number of CSP-specific CD4^+^ T cells per million (CMI) and adjuvant formulation or vaccination status.

|  | estimate | P value |
| --- | --- | --- |
| **vaccine cohort only** |  |  |
| *protection ~ log(IgG) + log (CMI) + adjuvant* | | |
| Intercept | -5.77 | 0.0018* |
| log(IgG) | 0.71 | 0.020* |
| log(CMI) | 0.35 | 0.035* |
| adjuvant | -0.22 | 0.659 |
|  |  |  |
| **vaccine and control cohorts** | | |
| *protection ~ log(IgG) + log (CMI) + vaccine* | | |
| intercept | -18.53 | 0.99 |
| log(IgG) | 0.75 | 0.0117* |
| log(CMI) | 0.36 | 0.0311* |
| vaccine | 12.42 | 0.99 |

**Sensitivity Analysis**

**Dependence of fixed parameters**

A number of parameters in the sporozoite infection model were estimated as fixed values derived from the literature, namely the latent period between sporozoite inoculation and emergence of merozoites from the liver (*t_L_* = 6.5 days) [[4](#_ENREF_4)], the daily multiplication rate of merozoite in the blood (*m* = 3.8 day^-1^) [[5](#_ENREF_5)], and the threshold parasite density at which infection is detectable by slide microscopy (*p_T_* = 50,000,000 parasites ⬄ 10,000 parasites/mL) [[6](#_ENREF_6)]. To investigate the dependence of model fitting results on these parameter estimates, we performed a sensitivity analysis varying each of the fixed parameters one at a time. The results are presented in Table S9. The choice of fixed parameters had no significant affect on the model estimates of immunological parameters or vaccine efficacy (β_ab_, α_ab_, β_cmi_, *VE_i_*, *VE_s_*) when compared to the 95% confidence intervals presented in Table 1. The choice of fixed parameters did however affect model estimates of the mean and variance of the numbers of sporozoites and merozoites.

Table S9: Sensitivity analysis demonstrating the effect on model estimated parameters and efficacy of varying fixed parameters one at a time. Estimated parameters that fell outside of the 95% confidence intervals of the baseline parameter set (*m*=3.8, *t_L_*=6.5, *p_T_*=5x10^7^) are highlighted in bold.

| **Fixed parameters** | | | **Estimated parameters** | | | | | | | **Estimated efficacy** | |
| --- | --- | --- | --- | --- | --- | --- | --- | --- | --- | --- | --- |
| ***m*** | ***t_L_*** | ***p_T_*** | ***n*** | **σ*_n_*** | **µ** | **σ_µ_** | **β_ab_** | **α_ab_** | **β_cmi_** | ***VE_i_*** | ***VE_s_*** |
| 3.8 | 6.5 | 5 x 10^7^ | 150 | 194 | 2136 | 4460 | 6.62 | 1.32 | 1367 | 40.0% | 96.1% |
| 3.8 | 6.0 | 5 x 10^7^ | 156 | 202 | **1060** | **2224** | 6.70 | 1.33 | 1368 | 40.0% | 96.2% |
| 3.8 | 7.0 | 5 x 10^7^ | **341** | **489** | 2313 | 5404 | 6.30 | 1.41 | 1328 | 36.7% | 96.8% |
| 3.0 | 6.5 | 5 x 10^7^ | 117 | 146 | **5800** | **9972** | 9.94 | 1.33 | 1572 | 34.5% | 94.1% |
| 4.5 | 6.5 | 5 x 10^7^ | **297** | **420** | **649** | **1530** | 5.70 | 1.41 | 1264 | 40.3% | 97.1% |
| 3.8 | 6.5 | 1 x 10^7^ | 128 | 163 | **500** | **1022** | 6.27 | 1.28 | 1392 | 41.2% | 96.1% |
| 3.8 | 6.5 | 1 x 10^8^ | **346** | **496** | 2349 | 5501 | 6.30 | 1.42 | 1327 | 36.6% | 96.8% |

**Distribution of parasite numbers**

In the sporozoite infection model, variation in blood-stage parasite numbers arises due to either variation in the number of successfully infecting sporozoites, or variation in the number of merozoties released per sprozoite. Although estimates derived from blood-stage parasite numbers will also be affected by variation in blood-stage parasite growth rates [[5](#_ENREF_5),[13](#_ENREF_13),[14](#_ENREF_14)], we assume constant growth rates due to a lack of data on repeated measurements of parasite density. We assume the number of infectious sporozoites follows a Negative Binomial distribution, and the number of merozoites per sporozoite follows a Gamma distribution. The estimated distributions are shown in Figure S1.

Note that the distribution for the number of sporozoites is heavily skewed, with most 5 bite mosquito challenges predicted to result in fewer than 50 successful sporozoites, and a small proportion predicted to result in greater than 300 sporozoites, in agreement with experimental observation [[1](#_ENREF_1),[2](#_ENREF_2)]. We assumed the number of merozoites released per sporozoite to be Gamma distributed with mean $\mu$and standard deviation $\sigma_{\mu}$. As our estimate of the standard deviation was greater than the mean the distribution is predicted to be skewed (Figure S1B). The number of merozoites released from *n* sporozoites will also be Gamma distributed with the distribution looking approximately Normal for large *n* (Figure S1C).

Figure S1: Estimated distributions of parasite numbers: (A) The number of sporozoites that survive liver stage development after challenge with 5 *P. falciparum* infectious mosquito bites follows a Negative Binomial distribution with mean 150 and standard deviation 194. (B) The number of merozoites released per sporozoite follows a Gamma distribution with mean µ = 2136 and standard deviation σ_µ_ = 4160. (C) The number of merozoites released from a batch of sporozoites will follow a Gamma distribution, with the distribution looking approximately Normal for large *n*.

The variation in the total liver-to-blood inocula, and hence the variation in time to onset of parasitemia, will depend on the variation in the number of infectious sporozoites and the variation in the number of merozoites per sporozoite (Figure S1). In order to investigate the relative contribution of both these variances, a sensitivity analysis was undertaken wherby the variance in the number of merozoites (σ_µ_) was fixed at a range of parameters and the rest of the parameters estimated using maximum likelihood estimation. For each set of parameters, the efficacy infection and efficacy per sporozoite were also calculated (Table S10). Although larger values of σ_µ_ resulted in smaller values of σ_n_, there were no substantial differences in the estimated vaccine efficacies.

Table S10: Dependence of maximum likelihood parameters and estimated efficacy against infection and vaccine efficacy per sporozoite on the variation in the number of merozoites per sporozoite σ_µ_.

| σ_µ_ | *n* | σ_n_ | µ | β_ab_ | α_ab_ | β_cmi_ | ML | *VE*_i_ | *VE*_s_ |
| --- | --- | --- | --- | --- | --- | --- | --- | --- | --- |
| 1000 | 181 | 266 | 1756 | 4.72 | 1.20 | 3141 | -1128.56 | 0.363 | 0.962 |
| 2000 | 176 | 249 | 1807 | 5.34 | 1.25 | 2268 | -1040.32 | 0.375 | 0.962 |
| 3000 | 170 | 228 | 1880 | 6.51 | 1.33 | 1553 | -1026.57 | 0.393 | 0.962 |
| 4000 | 160 | 208 | 2010 | 6.75 | 1.34 | 1378 | -1023.55 | 0.399 | 0.962 |
| 5000 | 135 | 172 | 2375 | 6.35 | 1.29 | 1380 | -1023.48 | 0.407 | 0.961 |
| 6000 | 114 | 141 | 2837 | 5.86 | 1.23 | 1413 | -1023.89 | 0.417 | 0.961 |
| 8000 | 86 | 104 | 3737 | 5.11 | 1.14 | 1482 | -1025.13 | 0.429 | 0.960 |
| 10000 | 70 | 82 | 4610 | 4.56 | 1.06 | 1545 | -1026.53 | 0.437 | 0.958 |

**References**

1. Beier JC, Davis JR, Vaughan JA, Noden BH, Beier MS (1991) Quantitation of Plasmodium-falciparum sporozoites transmitted invitro by experimentally infected Anopheles-gambiae and Anopheles-stephensi. American Journal of Tropical Medicine and Hygiene 44: 564-570.

2. Beier MS, Davis JR, Pumpuni CB, Noden BH, Beier JC (1992) Ingestion of Plasmodium-falciparum sporozoites during transmission by anopheline mosquitos. American Journal of Tropical Medicine and Hygiene 47: 195-200.

3. Medica DL, Sinnis P (2005) Quantitative dynamics of Plasmodium yoelii sporozoite transmission by infected anopheline mosquitoes. Infection and Immunity 73: 4363-4369.

4. Murphy JR, Baqar S, Davis JR, Herrington DA, Clyde DF (1989) Evidence for a 6.5-day minimum exoerythrocytic cycle for Plasmodium-falciparum in humans and confirmation that immunization with a synthetic peptide representative of a region of the circumsporozoite protein retards infection. Journal of Clinical Microbiology 27: 1434-1437.

5. Bejon P, Andrews L, Andersen RF, Dunachie S, Webster D, et al. (2005) Calculation of liver-to-blood inocula, parasite growth rates, and preerythrocytic vaccine efficacy, from serial quantitative polymerase chain reaction studies of volunteers challenged with malaria sporozoites. Journal of Infectious Diseases 191: 619-626.

6. Bejon P, Andrews L, Hunt-Cooke A, Sanderson F, Gilbert SC, et al. (2006) Thick blood film examination for Plasmodium falciparum malaria has reduced sensitivity and underestimates parasite density. Malaria Journal 5.

7. Schwenk RJ, Richie TL (2011) Protective immunity to pre-erythrocytic stage malaria. Trends in Parasitology 27: 306-314.

8. Vaughan JA, Scheller LF, Wirtz RA, Azad AF (1999) Infectivity of Plasmodium berghei sporozoites delivered by intravenous inoculation versus mosquito bite: Implications for sporozoite vaccine trials. Infection and Immunity 67: 4285-4289.

9. Moorthy V, Ballou WR (2009) Immunological mechanisms underlying protection mediated by RTS,S: a review of the available data. Malaria Journal 8:312.

10. Moore AC, Hutchings CL (2007) Combination vaccines: synergistic simultaneous induction of antibody and T-cell immunity. Expert review of vaccines 6: 111-121.

11. Greco WR, Bravo G, Parsons JC (1995) The search for synergy - a critical-review from a response-surface perspective. Pharmacological Reviews 47: 331-385.

12. White MT, Griffin JT, Riley EM, Drakeley CJ, Moormann AM, et al. (2010) Efficacy model for antibody-mediated pre-erythrocytic malaria vaccines. Proc Roy Soc B.

13. Hermsen CC, De Vlas SJ, Van Gemert GJA, Telgt DSC, Verhage DF, et al. (2004) Testing vaccines in human experimental malaria: Statistical analysis of parasitemia measured by a quantitative real-time polymerase chain reaction. American Journal of Tropical Medicine and Hygiene 71: 196-201.

14. Douglas AD, Andrews L, Draper SJ, Bojang K, Milligan P, et al. (2011) Substantially Reduced Pre-patent Parasite Multiplication Rates Are Associated With Naturally Acquired Immunity to Plasmodium falciparum. Journal of Infectious Diseases 203: 1337-1340.

15. Kester KE, Cummings JF, Ofori-Anyinam O, Ockenhouse CF, Krzych U, et al. (2009) Randomized, Double-Blind, Phase 2a Trial of Falciparum Malaria Vaccines RTS,S/AS01B and RTS,S/AS02A in Malaria-Naive Adults: Safety, Efficacy, and Immunologic Associates of Protection. Journal of Infectious Diseases 200: 337-346.

16. Prentice RL (1989) Surrogate endpoints in clinical-trials - definition and operational criteria. Statistics in Medicine 8: 431-440.

17. Qin L, Gilbert PB, Corey L, McElrath MJ, Self SG (2007) A framework for assessing immunological correlates of protection in vaccine trials. Journal of Infectious Diseases 196: 1304-1312.
